# Bacterial phagocytosis and reactive oxygen species production by camel neutrophils and monocytes are influenced by the type of anticoagulation agent

**DOI:** 10.14202/vetworld.2021.1888-1893

**Published:** 2021-07-24

**Authors:** Naser Abdallah Al Humam

**Affiliations:** Department of Microbiology, College of Veterinary Medicine, King Faisal University, Al-Ahsa, Saudi Arabia

**Keywords:** bacteria, dromedary camel, monocyte, neutrophil, phagocytosis, reactive oxygen species

## Abstract

**Background and Aim:**

Anticoagulants with different modes of action are used in the collection of camel blood samples. In the innate immune response, camel neutrophils and monocytes can play several roles during infection and inflammation. For anticoagulants ethylenediaminetetraacetic acid (EDTA) and heparin, research has described their effects on different parameters of the immune system. However, to date, no research has examined the effects of anticoagulants on the functional activity of camel phagocytes. Therefore, this study analyzed the influence of K_3_EDTA and lithium heparin on the antimicrobial activity of camel neutrophils and monocytes.

**Materials and Methods:**

Camel leukocytes were separated from blood collected in EDTA or heparin tubes, and their phagocytosis and reactive oxygen species (ROS) production activity were analyzed by flow cytometry after stimulation with *Staphylococcus* *aureus* or *Escherichia coli* bacteria.

**Results:**

In comparison to the cells collected from the EDTA blood, the camel neutrophils and monocytes separated from the heparin blood showed higher phagocytosis activity of *S. aureus* and *E. coli*. In addition, the neutrophils and monocytes produced significantly more ROS when the blood was collected in the heparin tubes.

**Conclusion:**

The antimicrobial functions of camel neutrophils and monocytes are significantly affected by the type of anticoagulation agent. Therefore, using heparin rather than EDTA as an anticoagulant is recommended when performing the functional analysis of phagocytosis and ROS production of camel phagocytes.

## Introduction

Camel (*Camelus dromedarius*) polymorphonuclear neutrophils and monocytes are key players in the immune system, having several roles during infection and inflammation [1]. As members of the phagocytosis system, neutrophils and monocytes contribute to the early elimination of bacterial pathogens by phagocytosis and the subsequent killing of microbes by various mechanisms [2].

Separated polymorphonuclear neutrophils and monocytes can be used in multiple experimental analyses depending on the scientific research question. In particular, the functional analysis of neutrophils and monocytes (e.g., respiratory burst activity and ­phagocytosis) has been found to be sensitive to blood collection and cell separation techniques [3]. For the collection of blood samples, anticoagulants with different modes of action have been used [4]. For example, ethylenediaminetetraacetic acid (EDTA), a well-known metal-chelating agent, prevents blood coagulation by chelating free calcium ions (Ca^2+^) in plasma [5]. The heparin anticoagulation effect depends on preventing the conversion of fibrinogen into fibrin [5]. For both anticoagulants, several effects on cell phenotype and function have been described previously [6-9].

Because no studies have been conducted on the effects of anticoagulants on the functional activity of camel phagocytes, this study analyzed the influence of the most frequently used anticoagulants K_3_EDTA and lithium heparin on the antimicrobial activity of blood neutrophils and monocytes from the dromedary camel.

## Materials and Methods

### Ethical approval

The Ethics Committee at King Faisal University, Saudi Arabia approved all experimental procedures and management conditions used in this study (Permission number: KFU- KFU-REC/2020-09-25).

### Study period and location

The study was conducted from October to December 2020 at the Camel Research Center, King Faisal University in Al-Ahsa Saudi Arabia.

### Animals and blood sampling

Blood samples were collected from 6 male camels (*Camelus dromedaries*), ages 10-13 years, housed at the farm of the Camel Research Center, King Faisal University, Al-Ahsa, Saudi Arabia. Blood was obtained by venipuncture of the *vena jugularis* *externa* into vacutainer tubes containing EDTA or lithium heparin (Becton Dickinson, Heidelberg, Germany). The collected blood samples were transported within 1 h to the laboratory for cell separation and flow cytometric analysis.

### Cell separation

Camel leukocytes were separated from the blood samples by hypotonic lysis of the red blood cells. The samples were incubated in distilled water for 20 s followed by the addition of double-concentrated phosphate-buffered saline (PBS) to restore tonicity. This hemolysis procedure was repeated twice until erythrolysis was completed. Separated leukocytes then were suspended in Roswell Park Memorial Institute medium at 6×10^6^ cells/mL. The mean viability of the separated cells was evaluated after labeling with propidium iodide (2 μg/mL; Calbiochem, Germany); it was >90%.

### Phagocytosis assay

Heat-killed *Staphylococcus aureus* or *Escherichia coli* bacteria were labeled with fluorescein isothiocyanate (FITC; Sigma-Aldrich, St. Louis, MO, USA). Separated leukocytes were plated in 96 well plates (1×10^5^/well) and incubated with FITC-labeled *S. aureus* or *E. coli* (40 bacteria/cell) for 30 min at 37°C and 5% carbon dioxide. To identify the monocytes, the cells were incubated with allophycocyanin (APC)-conjugated monoclonal antibodies specific to the cell surface molecule CD14 (clone tuk 4) [1].

After incubation (15 min at 4°C), the labeled cells were analyzed by flow cytometry. The phagocytosis activity of the monocytes and neutrophils was defined as the percentage of green fluorescing cells among the total cells. Mean fluorescence intensity (MFI) of the phagocytosis-positive cells was measured as an indicator for the number of bacteria phagocytosed by each cell.

### Generation of reactive oxygen species (ROS)

ROS generation was performed in 96-well, round-bottom microtiter plates (Corning, NY, USA) [10]. Leukocytes (1×10^6^/well) were incubated for 30 min (37°C, 5% CO_2_) with heat-killed *S. aureus* bacteria (30 bacteria/cell) in the presence of ROS-sensitive dye dihydrorhodamine (DHR)-123 (750 ng/mL final; Mobitec, Goettingen, Germany). For monocyte identification, the cells were incubated with APC-conjugated monoclonal antibodies specific to CD14. After incubation, the cells were washed with PBS, and the percentage of ROS-positive cells and the relative amount of generated ROS were determined by flow cytometry (Accurie C6 Flow Cytometer; BD Biosciences, USA) after the acquisition of 100,000 events.

### Statistical analysis

Statistical analysis was performed with GraphPad software version 5, (GraphPad Software, San Diego, USA). The results are presented as mean±standard error of the mean (SEM). Differences between means were tested with the Student’s t*-*test, and the results were considered significant if p<0.05.

## Results

### Impact of the anticoagulation agent on phagocytosis function of the neutrophils and monocytes

**Figure-1:**
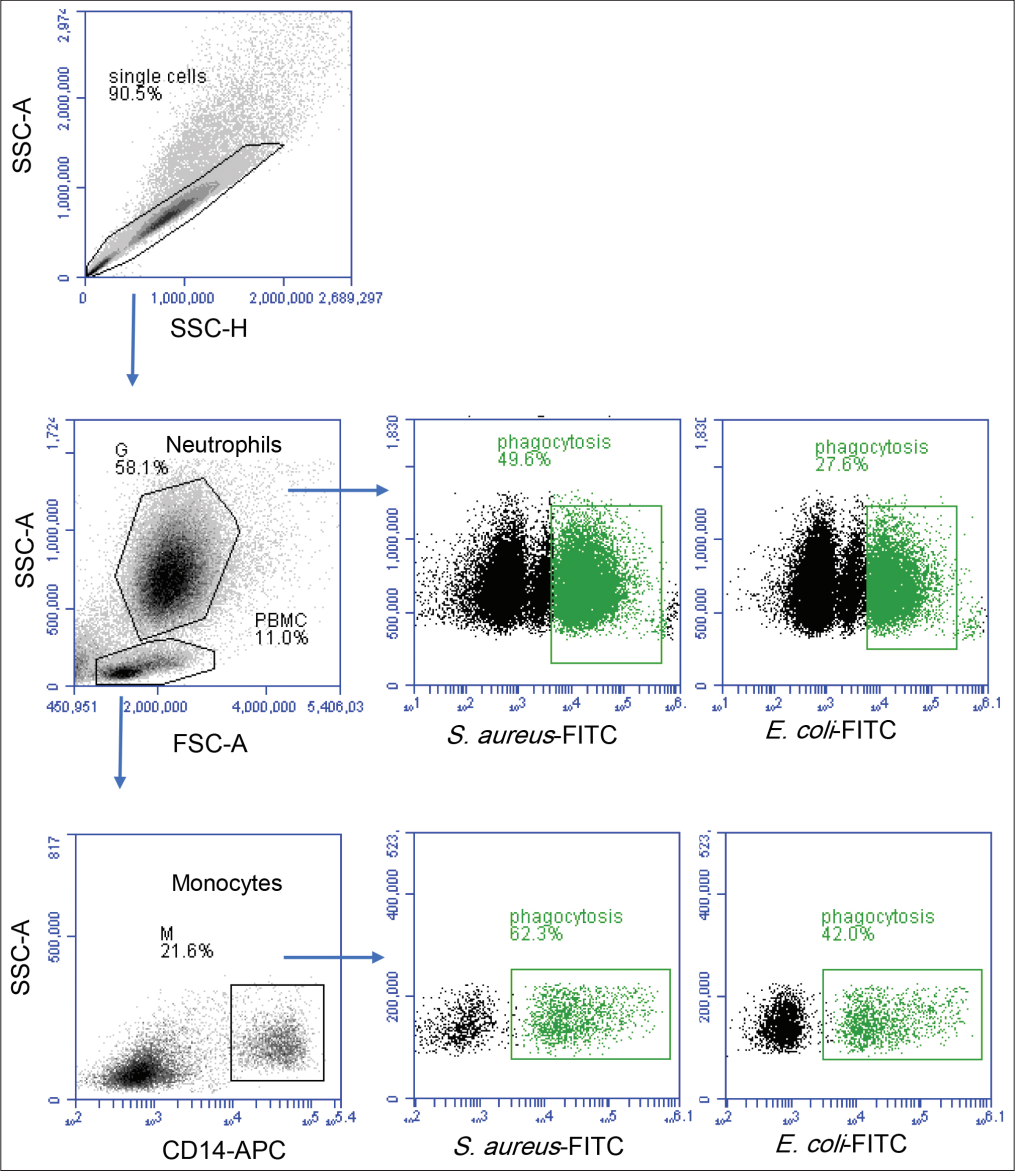
Flow cytometric analysis of bacterial phagocytosis by camel neutrophils and monocytes. Separated camel leukocytes were incubated with fluorescein isothiocyanate-labeled heat-inactivated *Staphylococcus aureus* or *Escherichia coli* and the labeled cells were analyzed by flow cytometry. Cell duplets were excluded from the analysis using an SSC-A/SSC-H dot plot. Camel granulocytes and monocytes were identified based on their FSC and SSC properties and positive staining with CD14, respectively. After gating on granulocytes and monocytes, phagocytosis-positive cells were defined based on their higher green fluorescence. The mean fluorescence intensity of phagocytosis-positive cells was also calculated.

For the analysis of the impact of the ­anticoagulant on the phagocytosis function of the blood phagocytes, the capacity of the camel neutrophils and monocytes to ingest the bacterial pathogens *S. aureus* and *E. coli* was analyzed using flow cytometry (Figure-1).

**Figure-2:**
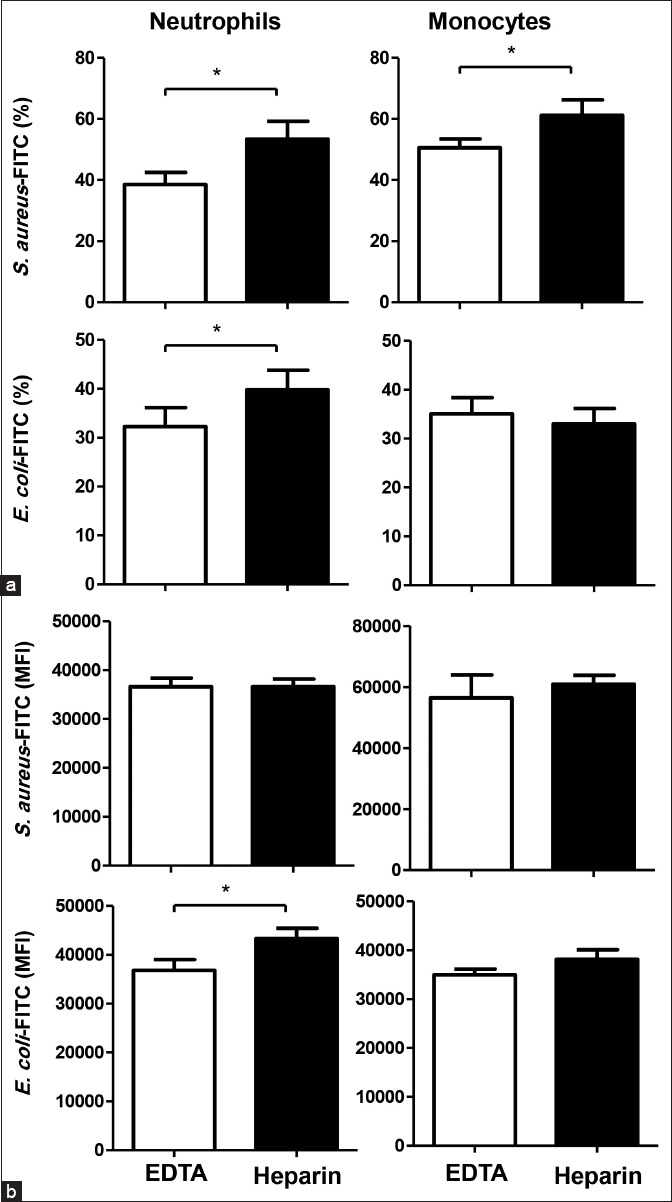
The impact of anticoagulants on the phagocytosis activity of camel neutrophils and monocytes. Separated camel leukocytes were incubated with fluorescein isothiocyanate-labeled heat-inactivated *Staphylococcus aureus* or *Escherichia coli* and the labeled cells were analyzed by flow cytometry. (a) The percentage of phagocytosis-positive cells within gated granulocytes and monocytes was calculated for samples collected in ethylenediaminetetraacetic acid (EDTA) and lithium heparin. (b) The mean fluorescence intensity of phagocytosis positive cells was also calculated for granulocytes and monocytes from samples collected in EDTA and lithium heparin. The data were presented graphically (means±standard error of the mean) (*p<0.05).

The percentage of the phagocytosis-positive cells within *S. aureus-*incubated neutrophils and monocytes was significantly higher (p<0.05) for cells separated from lithium heparin anticoagulated blood (53.4±5.7% for neutrophils and 61.2±5.0% for monocytes) as compared to those from EDTA-anticoagulated blood (38.6±3.9% for neutrophils and 50.5±2.9% for monocytes). However, only neutrophils (39.8±3.8% for heparin vs. 32.2±3.9% for EDTA) increased (p<0.05) their phagocytosis-positive fraction when the cells were incubated with *E. coli* (Figure-2a).

The analysis of phagocytosis capacity (MIF of phagocytosis-positive cells), as an indicator of the number of bacteria ingested by each cell, revealed a higher capacity for cells separated from lithium heparin blood in comparison to EDTA blood. The effect was, however, significant only for neutrophils and *E. coli* bacteria (43,315±2132 for heparin vs. 36,807±2194 for EDTA) (p<0.05) (Figure-2b).

### Impact of the anticoagulation agent on S. aureus-induced ROS production by neutrophils and monocytes

**Figure-3:**
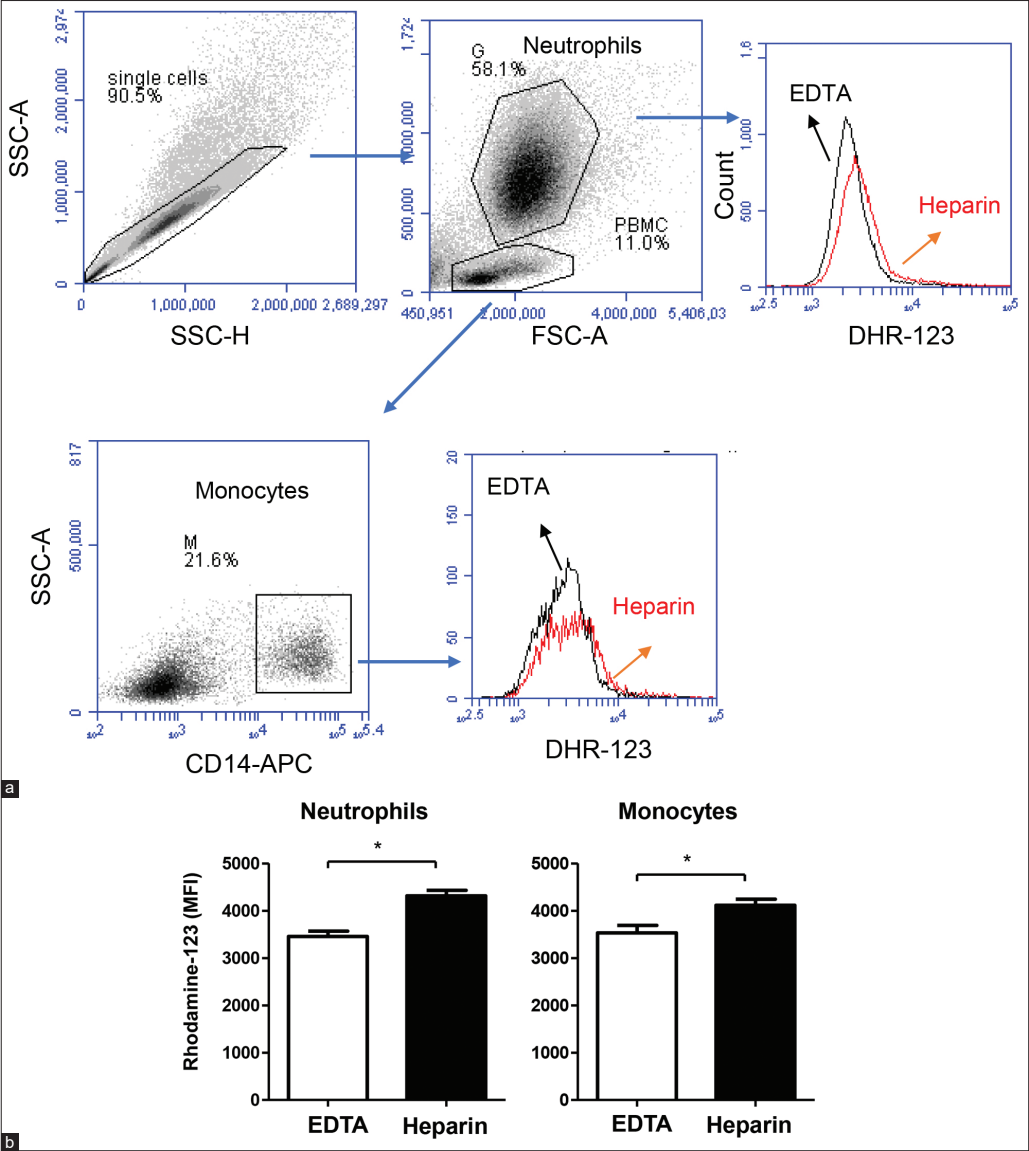
The impact of anticoagulant on *Staphylococcus aureus*-induced reactive oxygen species (ROS)-response of camel neutrophils and monocytes. (a) Flow cytometric analysis of ROS production by camel neutrophils and monocytes. Separated camel leukocytes were stimulated with heat-killed *Staphylococcus aureus* bacteria in the presence of the ROS-sensitive dye dihydrorohdamin-123 and the reactive oxygen-dependent generation of rhodamine-123 was analyzed by flow cytometry. After excluding cell duplicates and gating on neutrophils and monocytes, the mean fluorescence intensity (MFI) of rhodamine-123 was presented in a count/FL-1 histogram. (b) MFI values of rhodamine-123 were presented for granulocytes and monocytes (mean±standard error of the mean) from ethylenediaminetetraacetic acid and lithium heparin blood. The Student’s t-test was used for comparison between the means (*indicates p<0.05).

For ROS analysis, camel leukocytes were stimulated *in vitro* with heat-killed *S. aureus* bacteria, and the MFI of DHR-123 was analyzed with flow cytometry (Figure-3a). The amount of *S. aureus-*induced ROS production (mean±SEM of MFI) was significantly (p<0.05) higher for the neutrophils and monocytes separated from lithium heparin anticoagulated blood (4533±114 for neutrophils and 4114±129 for monocytes) than for cells from EDTA (3455±115 for neutrophils and 3533±156 for monocytes) anticoagulated blood (Figure-3b).

## Discussion

The blood phagocytes, neutrophils, and monocytes are key innate immune cells with central roles in antimicrobial defense against bacterial pathogens during early-stage infection [10-12]. Neutrophil and monocyte antimicrobial activity is mediated mainly by phagocytosis and the subsequent killing of microbes through oxygen-dependent and -independent mechanisms [13]. For the functional analysis of neutrophils and monocytes, blood samples usually are collected in tubes containing anticoagulation agents with different modes of action.

In the present study, using lithium heparin as an anticoagulant resulted in a higher percentage of phagocytosis-positive neutrophils and monocytes after incubation with *S. aureus* as compared to cells from EDTA-anticoagulated blood. Several studies have reported the requirement of Ca for effective phagocytosis [14]. EDTA prevents blood coagulation by chelating Ca^2+^ in plasma [5]. EDTA’s inhibitory effects on leukocyte phagocytosis activity have been reported in several species [5,15]. The reduced activity of the camel phagocytes separated from EDTA blood in comparison to heparin blood may be a result of the Ca-chelating effect of EDTA. However, when the leukocytes were stimulated with *E. coli*, only neutrophils from the EDTA blood decreased their phagocytosis-positive fraction as well as their capacity (number of bacteria ingested by each cell), in comparison to cells from the heparin-anticoagulated blood. Further investigations are required to determine, whether anticoagulant type is related to specific cell type or pathogen species modulatory mechanism.

This analysis of *S. aureus-*induced ROS revealed higher production for neutrophils and monocytes separated from lithium heparin than that for cells from EDTA-anticoagulated blood. Heparin-isolated bovine control granulocytes have shown more ROS production than that from EDTA-separated cells [5]. Enhanced ROS production is related to higher Ca^2+^ levels in granulocytes isolated from heparinized blood [16]. Similarly, in human neutrophils, EDTA has been shown to lead to a lower degree of phorbol myristate acetate-induced respiratory burst activation as compared to citrate and heparin [8]. Furthermore, the decreased ROS production ability of neutrophils and monocytes could be due to the reduced expression of phagocytosis receptors such as the complement receptor 3, which has been found to be expressed in lower quantities in cells from EDTA blood as compared to heparin blood [5,17]. However, a recent study has revealed no significant effects of anticoagulant type on the respiratory burst of human neutrophils [3].

## Conclusion

The antimicrobial functions of camel neutrophils and monocytes are significantly affected by the type of anticoagulation agent. For blood collected in lithium heparin, higher phagocytosis activity of *S. aureus* and *E. coli* occurred in the neutrophils and monocytes. In addition, both produced significantly more ROS when blood was collected in heparin tubes. Therefore, these results suggest using heparin rather than EDTA as an anticoagulant when performing the functional analysis of phagocytosis and ROS production for camel phagocytes.

## Author’s Contributions

NAA performed the experiments, prepared and revised the manuscript. NAA has read and approved the final manuscript.
